# Early Childhood Caries Prevalence and Associated Risk Factors in Monastir, Tunisia: A Cross-Sectional Study

**DOI:** 10.3389/fpubh.2022.821128

**Published:** 2022-02-25

**Authors:** Farah Chouchene, Fatma Masmoudi, Ahlem Baaziz, Fethi Maatouk, Hichem Ghedira

**Affiliations:** ^1^Pediatric and Preventive Dentistry Department, Faculty of Dental Medicine of Monastir, Monastir, Tunisia; ^2^Laboratory of Biological Clinical and Dento-Facial Approach (ABCDF Laboratory LR12ES10), University of Monastir, Monastir, Tunisia

**Keywords:** early childhood caries, prevalence, caries risk factors, Tunisia, epidemiological study

## Abstract

**Purpose:**

The present study aimed to investigate the prevalence and risk factors of ECC among preschool children in Monastir, Tunisia.

**Methods:**

The survey was designed as a cross-sectional study carried out between February and April 2021 in the main region of Monastir, Tunisia. A total of 381 preschool children were randomly selected using a three-stage clustered sampling technique from 10 daycares. The dental caries were diagnosed using WHO recommendations and a questionnaire in Arabic language was used to record personnel profile of the enrolled children. The chi-square test was used in bivariate analyses to assess the association between ECC and risk factors. Variables showing significant associations were included in multiple logistic regression models.

**Results:**

The prevalence of ECC was 20% and the mean dmft score was 0.89 ± 0.24. The prevalence of ECC increased at the age of 48–59 months ([OR] 2.602; 95%CI: 1.122–2.302), the age of 60–71 months ([OR] 2.845; 95% CI: 1.128–2.072), in children with nocturnal feeding ([OR] 2,417; 95% CI: 1.340–4,358), who take sugary drinks in the bottle ([OR] 1.104; 95% CI: 1.667–2.826), stopped breast or bottle feeding after the age of 18 months ([OR] 2.417; 95% CI: 1.340–4.358), do not brush their teeth properly ([OR] 1.435; 95% CI: 1.207–2.915), had visited a dentist ([OR] 2.444; 95% CI: 2.072–1.108), and decreased in children with a more highly educated parents ([OR] 0.797; 95%CI: 0.171–0.650).

**Conclusion:**

Given the relatively high prevalence of ECC in Tunisia, it is important to review public dental health policies and develop effective strategies to encourage changes in behavior related to the oral health of children to prevent the spread and worsening of this disease.

## Introduction

Early childhood caries (ECC) has become a significant health problem among children and infant. The American Academy of Pediatric Dentistry defined ECC as the presence of one or more carious (non-cavitad or cavitaded lesions), missing (due to caries), or filled tooth surfaces in one or more primary teeth in children aged 71 months or younger (1–3).

According to a systematic with meta-analysis published in 2021, ECC is considered as a global health problem, affecting about almost half, of preschool children. Sixty-four reports of 67 countries (published between 1992 and 2019) which covered 29 countries/59018 children showed that the ECC prevalence varied widely, and there was more variance attributable between-country differences rather than continent or change over time (4).

The prevalence by continent was Africa: 30 [19, 45]; Americas: 48 [42, 54]; Asia: 52 [43, 61]; Europe: 43 [24, 66]; and Oceania: 82 [73, 89]. Africa had a lower prevalence than the global pooled prevalence, whereas Asia, Oceania, and North and Central America showed a prevalence above the global estimate. Europe and America were within the global estimate. However, there was a lack of certainty as to the accuracy of the result, as many countries (and regions) were not represented such as Tunisia (4).

To describe ECC different terminologies were used such as; rampant caries, prolonged nursing habit caries, nursing bottle caries, nursing caries, baby bottle tooth decay, baby bottle caries, and milk bottle syndrome (5).

The terminologies of ECC reflect its multifactorial characteristics (6). In addition to the simultaneous interaction of factors including susceptible tooth surface/host, cariogenic microorganisms, and carbohydrates, epidemiological studies have documented transfer of cariogenic microorganisms from mother to her children, low socioeconomic status, gestational age, weight at birth, insufficient child oral health-care, unbalanced bottle-feeding, and bad brushing behaviors as ECC risk factors (5, 7).

ECC which is considered as a significant health issue reported among young children, may be associated with other health conditions, ranging from local pain, abscesses, to more severe problems leading to difficulty in chewing, malnutrition, and gastrointestinal disorders (8).

Further, left untreated, ECC may considerably influence the child quality of life, his self-esteem, his socializing and learning abilities (6, 9).

To optimize the chances of young children to stay free of early dental caries, it is mandatory to initiate preventive programs soon or after primary tooth eruption (9, 10).

Despite being preventable, ECC has remained relatively unexplored in many countries including Tunisia. Little work has been done on determining the prevalence of ECC among Tunisian preschool children and only a few studies have been published. According to Chamli et al. (11), the prevalence of ECC in Sousse, Tunisia was 45%. While in the study conducted by Maatouk et al. (12) the prevalence of dental caries among preschool children aged 3–5 years reported in the same region of Monastir in 2002 was 36%. Monitoring the prevalence of ECC in Tunisia is a key element in planning services and determining progress toward controlling this commonest dental disease in children. This study aimed to investigate the prevalence and risk factors of ECC among preschool children aged 3–5 years in Monastir, Tunisia.

## Materials and Methods

### Study Design and Sample

The present study was designed as a cross-sectional survey carried out between February and April 2021 in the main region of Monastir Tunisia.

Before initiation of the study, the sample size was calculated assuming a prevalence of ECC of about 45% (11) with a margin of error of 5% and a 95% of confidence level. Accordingly, a sample size of 381 was sought. To select the children, a three-stage cluster sampling technique was followed. This technique consisted first of selecting the district which was stratified into two urban and rural areas, then over the 35 preschools stratified by district, 10 were randomly selected. Finally, three kindergarten classes representing children aged 3, 4, and 5 were randomly selected from each kindergarten.

In the present study, only healthy children who do not have history of any diseases under 6 years of age (3–5 years) in primary dentition attending the previously selected kindergarten present at the time of the study and whose parents signed an informed consent were included.

Children in mixed teeth, absent during the dental examination and/or whose parents did not consent to their participation were excluded.

### Questionnaire

To record personnel profile of the children, a questionnaire in Arabic language was designed by the investigator according to the guidelines of the American Academy of Pediatric Dentistry (13, 14).

The questionnaire included information about; sociodemographic characteristics of the enrolled preschool children (gender, age, family size, birth order, family annual income, family health insurance, parent's age, and education level).

Children's gestational age, dietary behaviors (history of feeding, frequency of sweet and soft drinks consumption), oral hygiene behaviors (start of tooth brushing, daily brushing frequency, tooth brushing methods, parental supervision, using fluoride toothpaste), history of dental visit.

The questionnaire was distributed in each selected school with a covering letter for informed consent to all the children, and collected 2 week later after being completed by the parents. Responses were anonymous and participation was voluntary.

The investigator reviewed the questionnaires for appropriateness and children who fulfilled the inclusion criteria were examined.

### Clinical Examination

Using non-invasive technique and knee-to-knee posture in the kindergarten classes, the enrolled children underwent an oral examination which was performed by the same pediatric dentist (F.C). Two subjects from each school were re-examined on the same day as their initial dental examination to ensure the reproducibility of the application of diagnostic criteria between children. The intra-examiner agreement was 90%. No x-rays were performed. All the children were examined visually under natural light and. The dental caries were diagnosed using the World Health Organization (WHO) recommendations for oral health surveys (15). The “dmft index” by calculating the number of decayed (d), missing teeth (m), filled teeth (f), teeth (t) or surfaces (s), was used to assess the ECC.

In case of no evidence of treated or untreated clinical caries, the tooth was considered as sound.

A tooth was considered carious when there was an evident sign of cavity in a pit or fissure. A tooth with a temporary filling, or sealed but also decayed was recorded as carious. A tooth was considered filled when one or more permanent restorations were present and there was no caries. For missing teeth it was very important to differentiate those absent following the evolution of carious lesion from those lost due to physiological exfoliation (15).

### Ethical Consideration

The Preventive Dentistry Committee and the Faculty of Dental Medicine of Monastir approved the study. Permissions were obtained by the investigator from the Regional Delegation of the Ministry of Family and Child Welfare (Monastir, Tunisia) to visit the preschools establishments and to be able to examine the children. Informed consent of all the selected children's parents was obtained before the clinical examination.

### Statistical Analysis

The statistical analysis was performed using SPSS version 22.0 (IBM Corp., Windows, Armonk, NY, USA). To facilitate statistical analyses, some of the original variable were combined by the authors. Children age was grouped into; 36–47, 48–59, and 60–71 months. Parents education was categorized as; none/primary school, middle, high school, and university. Family income was grouped into: low, middle, and upper income according to the parents parents employments. Age stopped breast or bottle feeding was categorized as <12, 12–18, and >18 months. Tooth brushing methods were grouped intà; no particular method, vertical, horizontal tooth brushing and roll technique. The prevalence and mean dmft score were calculated to determine the extent of ECC in the study population. To measure the level of intra-examiner agreement and the reproducibility of the application of diagnostic criteria for dental caries, Cohen's Kappa was used (15). Frequencies and descriptive statistics were generated.

Bivariate analyses were performed to assess the association between ECC and risk factors using the chi-square test. Variables that showed significant associations were included in multiple logistic regression models. A *p* ≤ 0.05 was considered as statistically significant.

## Results

A total of 381 preschool children aged between 36 and 71 months with a mean age of 48 ± 9 months were included in the present survey. Of the participants, 49.9% were female and 50.1% were male. The ECC prevalence was 20% (*n* = 76) and the mean “dmft score” was 0.89 ± 0.24.

The prevalence of ECC and associated socioeconomic factors are shown in Table 1.

**Table 1:** Prevalence of ECC and associated socioeconomic factors (*N* = 381).

**Variables**	***N***	**%**	**Caries groups**		***p*-value**
			**Caries-free** **N (%)**	**ECC** **N (%)**	
**Gender**					
Girl	190	50.1	155 (50,8)	35 (46.1)	0.457
Boy	191	49.9	150 (49.2)	41 (53.9)	
**Age (months)**					
36–47	101	26.5	60 (19.6)	41 (53.9)	<0.001^*^
48–59	136	35.7	112 (36.7)	24 (31.6)	
60–71	144	37.8	133 (3.6)	11 (14.4)	
**Gestational age**					
Full-term	358	94	285 (93.4)	73 (96.1)	0.291
Premature birth	23	6	20 (6.6)	3 (3.9)	
**Family size**					
One child	174	45.6	142 (46.6)	32 (42.1)	0.352
More than one child	207	54.4	164 (79.2)	43 (20.8)	
**Birth order**					
1	171	44.9	141 (46.2)	30 (39.5)	0.330
2	137	36	107 (35.1)	30 (39.5)	
3	63	16.5	51 (16.7)	12 (15.8)	
4	9	2.4	5 (1.6)	4 (5.3)	
5	1	0.3	1 (0.3)	00 (00)	
**Mother's age**					
20–30	90	23.6	72 (23.6)	18 (23.7)	0.270
31–40	257	67.5	205 (67.2)	52 (68.4)	
>41	34	8.9	28 (9.2)	6 (7.9)	
**Father's age**					
20–30	29	7.6	25 (8.2)	4 (5.3)	0.070
31–40	212	55.6	200 (65.6)	12 (15.8)	
>41	140	36.8	80 (26.2)	60 (78.9)	
**Parents educational level**					
None/primary school	15	3.9	12 (4)	3 (3.9)	0.041^*^
Middle school	32	8.4	26 (8.5)	6 (7.9)	
High school	87	22.8	60 (19.7)	27 (35.5)	
University	247	64.8	207 (67.9)	40 (52.6)	
**Family annual income**					
Low income	128	33.5	98 (32.1)	30 (39.5)	0.316
Middle income	65	17.1	54 (17.7)	11 (14.5)	
Upper income	188	49.3	153 (50.2)	35 (46.1)	
**Parent's marital status**					
Married	372	97.6	300 (98.4)	72 (94.7)	0.060
Divorced	1	0.3	00 (00)	1 (0.3)	
Widowed	8	2.1	5 (1.6)	3 (3.9)	
**Family health insurance**					
Yes	361	94.8	290 (95.1)	71 (93.4)	0.367
No	20	5.2	15 (4.9)	5 (6.6)	

* *p ≤ 0.05*.

*Chi-square test*.

About 67.9% of caries-free children parents went to the university and 50.2% of them were issued from high socio-economic level. Univariate analyses showed a statistically significant relationship between ECC, children's age (*p* < 0.001) and parents' educational level (*p* = 0.041) (Table 1).

Table 2 shows the association of ECC with feeding history and dietary variables. Half of ECC-children took sugary drinks in bottle at night and 44.7% of them were breastfed for more than 18 months. Results showed a statistically significant association was found between ECC and the following variables; nocturnal feeding (*p* = 0.003), sugary drinks in bottle at night (*p* = 0.020), water in bottle during the day or the night (*p* = 0.030), and age stopped breast or bottle feeding (*p* = 0.027) (Table 2).

**Table 2:** Prevalence of ECC and associated feeding history/dietary habits (*N* = 381).

**Variables**	***N***	**%**	**Caries groups**		***p*-value**
			**Caries-free** **N (%)**	**ECC** **N (%)**	
**Feeding history**					
**Feeding type**					
Breast only	136	35.7	108 (35.4)	28 (36.8)	0.995
Breast and bottle	204	53.5	164 (53.8)	40 (52.6)	
Bottle only	41	10.8	33 (10.8)	8 (10.5)	
**Nocturnal feeding**					
No	66	17.3	44 (14.4)	22 (28.9)	0.003^*^
Yes	315	8.7	261 (85.6)	54 (71.1)	
**Sugary drinks in bottle at night**					
Yes	173	45.4	137 (44.9)	36 (47.4)	0.020^*^
No	208	54.6	168 (55.1)	40 (52.6)	
**Water in bottle (day/night)**					
Yes	105	27.6	91 (29.8)	14 (18.4)	0.030^*^
No	276	72.4	214 (70.2)	62 (81.6)	
**Age stopped breast-feeding/bottle**					
<12 months	113	29.7	86 (28.2)	27 (35.5)	0.027^*^
12–18 months	94	24.7	79 (25.9)	15 (19.8)	
>18 months	174	54.6	140 (45.9)	34 (44.7)	
**Current dietary habits**					
**Frequency of sweet and soft drinks consumption**					
Never	29	7.6	25 (8.2)	4 (3.3)	0.519
Once a day	208	54.6	170 (55.7)	38 (50)	
Twice a day	102	26.8	78 (25.6)	24 (31.6)	
More than twice a day	42	11	32 (10.5)	10 (13.2)	

* *p ≤ 0.05*.

*Chi-square test*.

There was a significant association between ECC prevalence, age of start tooth brushing (*p* = 0.008), tooth brushing frequency and methods (*p* = 0.041, *p* = 0.009), and parental supervision during the tooth brushing (*p* = 0.017) as reported in Table 3.

**Table 3:** Prevalence of ECC and oral hygiene behaviors/dental history (*N* = 381).

**Variables**	***N***	**%**	**Caries groups**		***p*-value**
			**Caries-free** **N (%)**	**ECC** **N (%)**	
**Start tooth brushing**					
≤ 3 years	71	18.6	51 (16.7)	20 (26.3)	0.008^*^
>3 years	310	81.4	254 (83.3)	56 (73.7)	
**Daily brushing frequency**					
No or irregular	9	2.4	7 (2.3)	2 (2.6)	0.041^*^
Once a day	208	54.6	170 (55.7)	38 (50)	
Twice a day	127	33.3	101 (33.1)	26 (34.2)	
More than twice a day	37	9.8	27 (8.8)	10 (13.2)	
**Tooth brushing methods**					
No particular method	102	26.7	88 (28.9)	14 (18.4)	0.009^*^
Vertical tooth brushing	145	38	112 (36.7)	33 (43.4)	
Roll technique	23	6	12 (3.9)	11 (14.5)	
Horizontal tooth brushing	111	29.1	93 (30.5)	18 (23.7)	
**Parental supervision**					
Yes	109	28.6	83 (27.2)	26 (34.2)	0.017^*^
No	272	71.4	222 (72.8)	50 (65.8)	
**Fluoride toothpaste**					
Yes	147	61.4	116 (38)	31 (40.8)	0.376
No	234	38.6	189 (62)	45(59.2)	
**History of dental visit**					
Yes	49	12.8	23 (7.5)	26 (34.2)	<0.001^*^
No	332	87.2	282 (92.5)	50 (65.8)	

* *p ≤ 0.05*.

*Chi-square test*.

A statistically significant association was found between ECC and history of dental visits (*p* < 0.001). Variables identified as being statistically significant in univariate analysis were entered into logistic regression models as shown in Table 4.

**Table 4:** Factors associated with ECC in multiple logistic regression analysis.

**Variable**	**OR**	**95% CI**		***X^**2**^***	***P-value***
		**Lower**	**Upper**		
Age (months)					<0.001
Age 1	2.602	1.122	2.302	42.117	<0.001
Age 2	2.845	1.128	2.072	34.379	<0.001
Parents educational level (reference, none/or primary school)	0.797	0.171	0.650	15.630	0.040
Nocturnal feeding (reference, no)	2,417	1.340	4,358	13.350	0.003
Sugary drinks in bottle at night (reference, no)	1.104	1.667	2.826	12.817	0.009
Water in bottle (day/night) (reference, no)	0.531	0.283	0.997	3.971	0.064
Age stopped breast-feeding/bottle (reference, >18 months)	2.417	1.340	4.358	40.336	0.003
Start tooth brushing (reference, ≤ 3 years)	0.836	0.504	1.385	10.230	0.440
Daily brushing frequency (reference, no or irregular)	0.503	0.069	1.684	3.189	0.503
Tooth brushing methods (reference, no particular method)	1.435	1.207	2.915	15.430	0.015
Parental supervision (reference, no)	1.385	0.809	1.369	4.801	0.061
History of dental visit (reference, no)	2.444	1.108	2.072	44.494	<0.001

*Age code: 36–47 months (0, 0); 48–59 months (1, 0); 60–71 months (0, 2)*.

*SE, Standard error; OR, Odds ratio; CI, Confidence interval; X^2^, Chi-square test value*.

Table 4 summarizes risk factors associated with ECC in multiple logistic regression analysis. Age of 48–59 months (odds ratio [OR] 2.602; 95% CI: 1.122–2.302), and age of 60–71 months (OR 2.845; 95% CI: 1.128–2.072) were significantly associated with greater odds of having ECC. Children with more highly educated parents were less likely to have ECC (OR 0.797; 95% CI: 0.171–0.650). Further, children with nocturnal feeding (OR 2,417; 95% CI: 1.340–4,358), who take sugary drinks in the bottle (OR 1.104; 95% CI: 1.667–2.826) and stopped breast or bottle feeding after the age of 18 months (OR 2.417; 95% CI: 1.340–4.358) were more likely to present ECC. Additionally, children who do not brush their teeth properly (OR 1.435; 95% CI: 1.207–2.915) and had visited a dentist (OR 2.444; 95% CI: 2.072–1.108) were more likely to present ECC, and no association was found with start tooth brushing age, daily brushing frequency and parental supervision.

## Discussion

In the present study, the prevalence of ECC among 3–5 years preschool children living in Monastir, Tunisia was 20% with a mean dmft score of 0.89 ± 0.24.

Even though dental caries remains a serious and important health problem among children, ECC is still relatively unexplored in Tunisia; only a few studies were conducted among preschool Tunisian children. According to Chamli et al. (11), the prevalence of ECC in Sousse Tunisia was 45% which was higher than the prevalence reported in the present survey; while in the study conducted by Maatouk et al. (12) the prevalence of dental caries among preschool children aged 3–5 years reported in the same region of Monastir in 2002 was 36%, which revealed a slight decrease in the dmft score over the past 20 years. These changes may be attributed to evolution in ECC associated risk factors over the time, the disparities of the studies samples and the fact that the studies were conducted in subgroups of the population. Although the number of children examined can be considered adequate, the low number of cavities reported in our study may be due to the parent's behavior and awareness of the importance of dental prevention.

According to a systematic review aiming to review the determinants of dental caries in children residing in the Middle East and North Africa (MENA) region, results including 94,491 participants in 14 countries, showed that the prevalence of ECC ranged between 3 and 57% (16). This wide variation between countries suggest that the distribution of ECC is not homogeneous and could be explained by genetic factors, ethnicity, and differences in socio-economic status (4).

In Casablanca, Morocco, the prevalence of ECC and severe-ECC were 74.2 and 47.3%, respectively (17). Results of this study suggests also that ECC negatively impacts the life quality of Moroccan Children in addition to their parents.

A previous narrative review by Tinanoff et al. based on 72 articles estimated a prevalence of ECC, across countries, of between 17% in France and 98% in Australia (18).

Recently published studies have shown that ECC was more widespread and frequent in countries that are underdeveloped and/or less developed, as well as among minorities living in well-developed countries (19).

According to some recent studies, the increasing tendency toward ECC may be associated with a rapid decline in the standard of living, a therapeutic approach in the resolution of the disease and certain demographic, psychosocial and behavioral characteristics specific to the region, which could modify the biological basis of the disease still insufficiently studied (19–22).

The fact that other published studies did not use the WHO criteria, included other diagnostic criteria, target population or examiner calibration may explain the decreased prevalence of ECC reported in our study (11, 12).

Due to the differences in the studies design, and other variables, comparing the present study findings to previous global and national studies was difficult.

The interaction of different etiological factors concurrently present initiate dental caries development. The known factors are essentially, the presence of cariogenic microorganisms, fermentable carbohydrates (substrate), and susceptible tooth surface/host.

In association to these well-demonstrate factors, a multitude of risk factors associated with ECC have been also reported the last decade.

In fact, epidemiological studies have documented other associated risk factors such as transfer of microbes from mother to child, low socioeconomic status, minority status, and low birth weight. In the developed countries, about 1–12% of children younger than 6 years experience ECC (21, 23).

Feeding, cariogenic food, child oral health-care and cleaning behaviors have been associated with ECC among children.

In the present study, children with nocturnal feeding, who take sugary drinks in the bottle and stopped breast or bottle feeding after the age of 18 months, are more likely to experience dental caries.

Regarding breast tor bottle feeding factors, some studies have reported that nighttime (20), long periods of breastfeeding and bottle feeding after the age of 18 months (24) were risk factors of ECC, while other studies have reported that nighttime feeding (breast and bottle feeding) after 12 months of age increased the risk of developing dental caries (25).

Although breastfeeding for at least 24 months is strongly recommended (26), when it is done on demand or especially at night with an associated high sugary diet and a late introduction of brushing, it can contribute toward high ECC (27).

In Tunisia, a study carried out among pediatricians showed that the majority of them recommend breastfeeding for at least 2 years and showed that 84% of them did not believe that nighttime breastfeeding is a risk factor for ECC, which may explain the implication of this factor in the development of caries lesions in the present study (28).

Additionally, it has been shown that during sleep, salivary flow decreases significantly thereby reducing the liquid carbohydrates clearance from the oral cavity, acting as a determining factor in the initiation of dental caries. It is therefore very important to reduce night-time breastfeeding, and sugary drinks in the bottle as much as possible and provide dentist early visit for examination and preventive advice concerning feeding practices (29).

However, in children who take water in their bottles, the prevalence of ECC was lower, which can be explained by the washing effect of the water or by the level of fluorides contained in the bottled water consumed by Tunisians. A same result was also reported in a study conducted by Al and Marshad et al. in Riyadh, Saudi Arabia (20).

In the present study, a statistically significant associations were reported between ECC prevalence, age of start tooth brushing, tooth brushing frequency and methods and parental supervision during the tooth brushing which was in accordance with numerous other studies carried out in different countries (11, 20, 22, 24, 25, 30).

According to a systematic review performed to assess the effect of tooth brushing frequency, individuals who report brushing their teeth infrequently have an increased risk of developing new carious lesions than those brushing more frequently, and this effect of brushing was more pronounced in primary than in permanent dentition (31).

Additionally, parents play an important role in their children's oral hygiene practices. According to a cross-sectional survey performed in Japan, lack of parental supervision was considered as a risk factor for dental caries in 6- to 7-year-old children (32).

Several studies (31, 33, 34), have reported a wide range, 9–72%, of young children, aged 1–5 years, brushing their teeth without their parents assistance. According to Zeedyk et al. (35) when young children are left on their own to brush their teeth, active tooth brushing may takes only an average of 10 s.

Parental supervision is not only about ensuring effective plaque removal through appropriate brushing technique, but also about supervising the amount of toothpaste used by children while brushing to reduce the incidence of fluorosis (33).

As reported in other studies a significant decrease of ECC was reported in children with a more highly educated parents (22, 36, 37).

In these studies it was reported that maternal education significantly influences attitudes toward children's oral health and that mothers play an important role in the development of children's dental health behavior (22, 36, 37). Moreover, parental practice such as feeding style and general parenting style can be relevant (32). According to a study conducted in Ohio, USA, permissive parenting style was more associated with dental caries than authoritative parenting style (38).

Results of the present study showed a statistically significant association between ECC and history of dental visits (*P* < 0.001) and children who visited a dentist during their infancy had a higher ECC prevalence. This is consistent with previous reports of an association between dentist visit and ECC in children (20, 39). This might reflect the fact that most children see the dentist only for an existing dental problem and not for prevention or control, and that parents in Tunisia believe that children do not need to be taken to the dentist unless they have a dental problem or pain caused by a carious lesion (20).

It is very important to highlight the significance of assessing the prevalence of dental caries in primary dentition, because according to recent research carious lesions in primary dentition may be used as a risk indicator for predicting not only dental caries but also enamel defects in their permanent successors (10, 40).

Since only a few studies about ECC have been conducted in Tunisia, the present results are considered as important but must be interpreted considering some limitations of the study. The sample size was slightly reduced, and only children enrolled in preschools were examined so the results may not be generalizable to children of the same age group not enrolled in preschools. In addition, as the caregiver data were retrospective, the possibility of some response bias could not be excluded. Nonetheless, the same findings may help to develop better prevention strategies.

Oral health programs should be established in Tunisia, focusing mainly on mothers, preschool teachers as well as young children.

It is also very important to start screening for ECC from early age; from the eruption of the first primary tooth or at the latest at the age of 1 year. To support oral health programs for children, health workers need to collect multiple epidemiological data through other surveys carried out in different regions of the country.

Awareness plays a very important role in the prevention of caries disease, which is why it is essential to sensitize health workers, in particular pediatricians, doctors, nurses and midwives, to the diagnosis, to prevention and treatment of childcare centers. The prevention of ECC in young children should begin with providing preventive dental care (application of fluoride varnishes/gel, crack sealants).

Parents, especially mothers, must be sensitized and educated in order to be able to help reducing the prevalence of ECC and this especially through the recognition of the first signs of ECC, good nutrition, supervision of tooth brushing, and cautious use sources of fluoride in high carious risk children. It is also important to underline the role of oral health promotion and prevention programs during pregnancy among future mothers. There is also a need to incorporate oral health education and motivation interventions in the pre and postnatal care programs in the Tunisian hospitals and public clinics (41).

## Conclusion

Findings of the present study demonstrate that dental caries in preschool children remain a serious oral health problem in Monastir. Parents educational level, poor oral hygiene, risky dietary, feeding behavior, and visits of a dental service, were associated with a higher risk of dental caries. It is therefore important to acknowledge that this risk may be reduced since all the described factors could be modified by improving Tunisian public health strategies, developing preventive strategies for primary dentition, providing health education for a large public and promoting the role of dentists in oral health promotion and prevention.

## Data Availability Statement

The raw data supporting the conclusions of this article will be made available by the authors, without undue reservation.

## Ethics Statement

The study was reviewed and approved by the Preventive Dentistry Committee and the Regional Delegation of the Ministry of Family and Child Welfare (Monastir, Tunisia). Written informed consent to participate in this study was provided by the participants' legal guardian.

## Author Contributions

FC conceived the idea, collected the data, and wrote the article. FC, FMas, and AB analyzed the data. FMaa and HG provided comprehensive judgement and assisted in editing the final version of the manuscript. All authors read and approved the final version of the manuscript prior to submission.

## Conflict of Interest

The authors declare that the research was conducted in the absence of any commercial or financial relationships that could be construed as a potential conflict of interest.

## Publisher's Note

All claims expressed in this article are solely those of the authors and do not necessarily represent those of their affiliated organizations, or those of the publisher, the editors and the reviewers. Any product that may be evaluated in this article, or claim that may be made by its manufacturer, is not guaranteed or endorsed by the publisher.
